# Case Report: Reconstruction of a Large Maxillary Defect With an Engineered, Vascularized, Prefabricated Bone Graft

**DOI:** 10.3389/fonc.2021.775136

**Published:** 2021-12-06

**Authors:** Tarek Ismail, Alexander Haumer, Alexander Lunger, Rik Osinga, Alexandre Kaempfen, Franziska Saxer, Anke Wixmerten, Sylvie Miot, Florian Thieringer, Joerg Beinemann, Christoph Kunz, Claude Jaquiéry, Thomas Weikert, Felix Kaul, Arnaud Scherberich, Dirk J. Schaefer, Ivan Martin

**Affiliations:** ^1^ Department of Plastic, Reconstructive, Aesthetic and Hand Surgery, University Hospital Basel, Basel, Switzerland; ^2^ Department of Biomedicine, University Hospital Basel, University of Basel, Basel, Switzerland; ^3^ Center for Musculoskeletal Infections, University Hospital Basel, Basel, Switzerland; ^4^ Department of Orthopedic Surgery, University Hospital Basel, Basel, Switzerland; ^5^ Clinic for Craniomaxillofacial and Oral Surgery, University Hospital Basel, Basel, Switzerland; ^6^ Department of Radiology, University Hospital Basel, Basel, Switzerland

**Keywords:** complex 3D bone defect, vascularized composite graft, bone–soft tissue interface, regenerative surgery, graft prefabrication

## Abstract

The reconstruction of complex midface defects is a challenging clinical scenario considering the high anatomical, functional, and aesthetic requirements. In this study, we proposed a surgical treatment to achieve improved oral rehabilitation and anatomical and functional reconstruction of a complex defect of the maxilla with a vascularized, engineered composite graft. The patient was a 39-year-old female, postoperative after left hemimaxillectomy for ameloblastic carcinoma in 2010 and tumor-free at the 5-year oncological follow-up. The left hemimaxillary defect was restored in a two-step approach. First, a composite graft was ectopically engineered using autologous stromal vascular fraction (SVF) cells seeded on an allogenic devitalized bone matrix. The resulting construct was further loaded with bone morphogenic protein-2 (BMP-2), wrapped within the latissimus dorsi muscle, and pedicled with an arteriovenous (AV) bundle. Subsequently, the prefabricated graft was orthotopically transferred into the defect site and revascularized through microvascular surgical techniques. The prefabricated graft contained vascularized bone tissue embedded within muscular tissue. Despite unexpected resorption, its orthotopic transfer enabled restoration of the orbital floor, separation of the oral and nasal cavities, and midface symmetry and allowed the patient to return to normal diet as well as to restore normal speech and swallowing function. These results remained stable for the entire follow-up period of 2 years. This clinical case demonstrates the safety and the feasibility of composite graft engineering for the treatment of complex maxillary defects. As compared to the current gold standard of autologous tissue transfer, this patient’s benefits included decreased donor site morbidity and improved oral rehabilitation. Bone resorption of the construct at the ectopic prefabrication site still needs to be further addressed to preserve the designed graft size and shape.

## Introduction

Reconstruction of large bone defects in the maxillofacial region, typically relying on autologous vascularized bone grafts or synthetic biocompatible materials, remains a clinical challenge. Apart from reconstructing the hard and soft tissues, the masticatory rehabilitation of the patient by conventional (removable) or implant-supported prostheses needs to be addressed. Autologous bone grafting is associated with limited availability, significant donor site morbidity, and restrained or even impossible oral rehabilitation. Synthetic materials represent only a temporary solution, not only because of frequency of infections but also due to limited integration and thus inadequate separation of oral and nasal cavities (1–3).

The potential for *de novo* bone formation of cells from the stromal vascular fraction (SVF) of adipose tissue, even if not cultured or primed *in vitro*, has been shown in preclinical and clinical models, if implanted orthotopically (4) or exposed to low doses of bone morphogenetic protein (BMP)-2 (5, 6). Moreover, efficient vascularization of critically sized, SVF-based bone grafts was achieved in a rodent model by the insertion of arteriovenous (AV) bundles (7, 8) by analogy with typical microsurgical techniques (9, 10).

Here we describe the pioneering clinical implementation of an ectopically prefabricated (i.e., including a vascular pedicle for transfer) and prelaminated (i.e., multilayer composite including a soft tissue interface) (11) flap as osteogenic and vasculogenic graft for hemimaxillary reconstruction in a patient with Cordeiro type IIIa maxillectomy. The implant was first constructed in a latissimus dorsi muscle flap by combining a custom-shaped scaffold with autologous SVF cells, BMP-2, and an AV bundle. The resulting prevascularized composite graft was then transferred to the maxilla defect with the ultimate goal to durably restore midface symmetry, separate naso- and oropharyngeal spaces, allow physiological swallowing, and establish airway function.

## Materials and Methods

**Figure 1:**
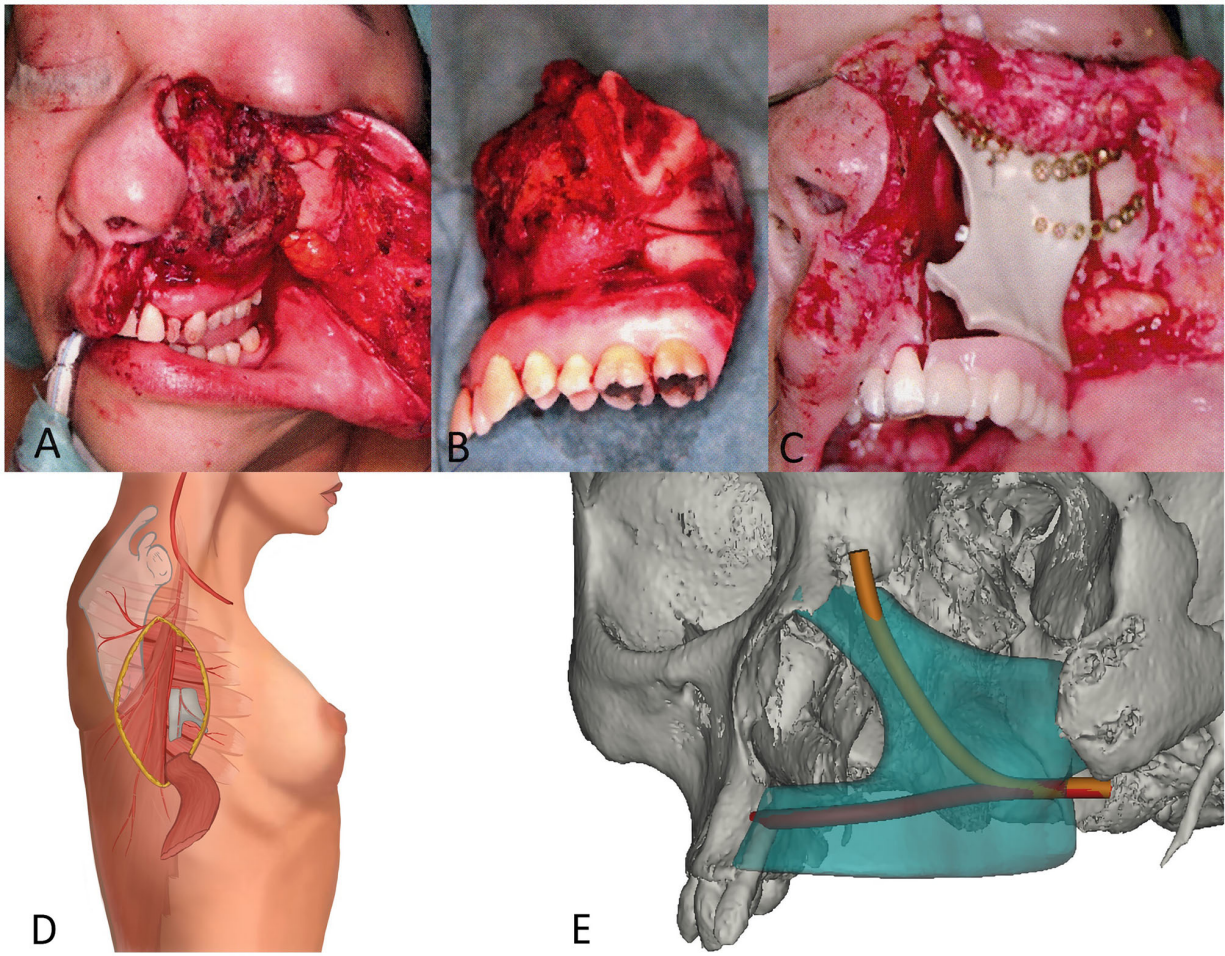
Midface defect, immediate temporary reconstruction, and two-step patient specific planning. **(A)** Intraoperative aspect of exposed ameloblastic carcinoma of the left hemimaxilla prior to resection. **(B)** Left hemimaxillectomy specimen with preservation of midface soft tissue and missing inner nasal lining (mucosa). **(C)** Intraoperative situation after PEEK implantation with obturator prosthesis. **(D)** Ectopic prevascularization by surgical insertion of an arteriovenous (AV) bundle (serratus branch of the thoracodorsal vessel) and wrapping of the construct into a split latissimus muscle. **(E)** CAD-CAM reconstruction on the patients CT scan showing the customized Tutoplast^®^ scaffold with the tunnel planned for the serratus AV bundle and its branching after implantation.

In this study, we report the case of a 39-year-old female patient diagnosed with an ameloblastic carcinoma of the left maxilla in September 2010. She underwent subsequent hemimaxillectomy in October 2010, which resulted in a Cordeiro type IIIa (total maxillectomy defect sparing the orbital contents) (12, 13), Okay Type II (14), Brown Type 3 (15) maxillectomy defect. The defect was replaced with a palatal obturator prosthesis (**Figures 1A–C**), which gave an unsatisfactory functional result. The orbital floor has been reconstructed with a titanium mesh (Synthes) during primary ameloblastoma resection. The patient suffered from recurrent infections and had problems drinking water due to insufficient separation of the oral and nasal cavity. After a 5-year cancer-free follow-up, the patient needed adequate and long-term surgical reconstruction of the left maxilla. The complexity of the patient’s three-dimensional composite defect, in combination with her young age and her desire to bypass morbidity and limited effectiveness of a free vascularized autologous bone graft (e.g., fibula, scapula or the iliac crest), prompted for the implementation of an innovative strategy. The patient had no other relevant comorbidities. This case study conforms with the Declaration of Helsinki. It was approved by the national competent authority, Swissmedic, under exceptional permission (SBH 16-0172) and by the competent ethical committee (EKNZ), with the patient’s written informed consent.

**Figure 2:**
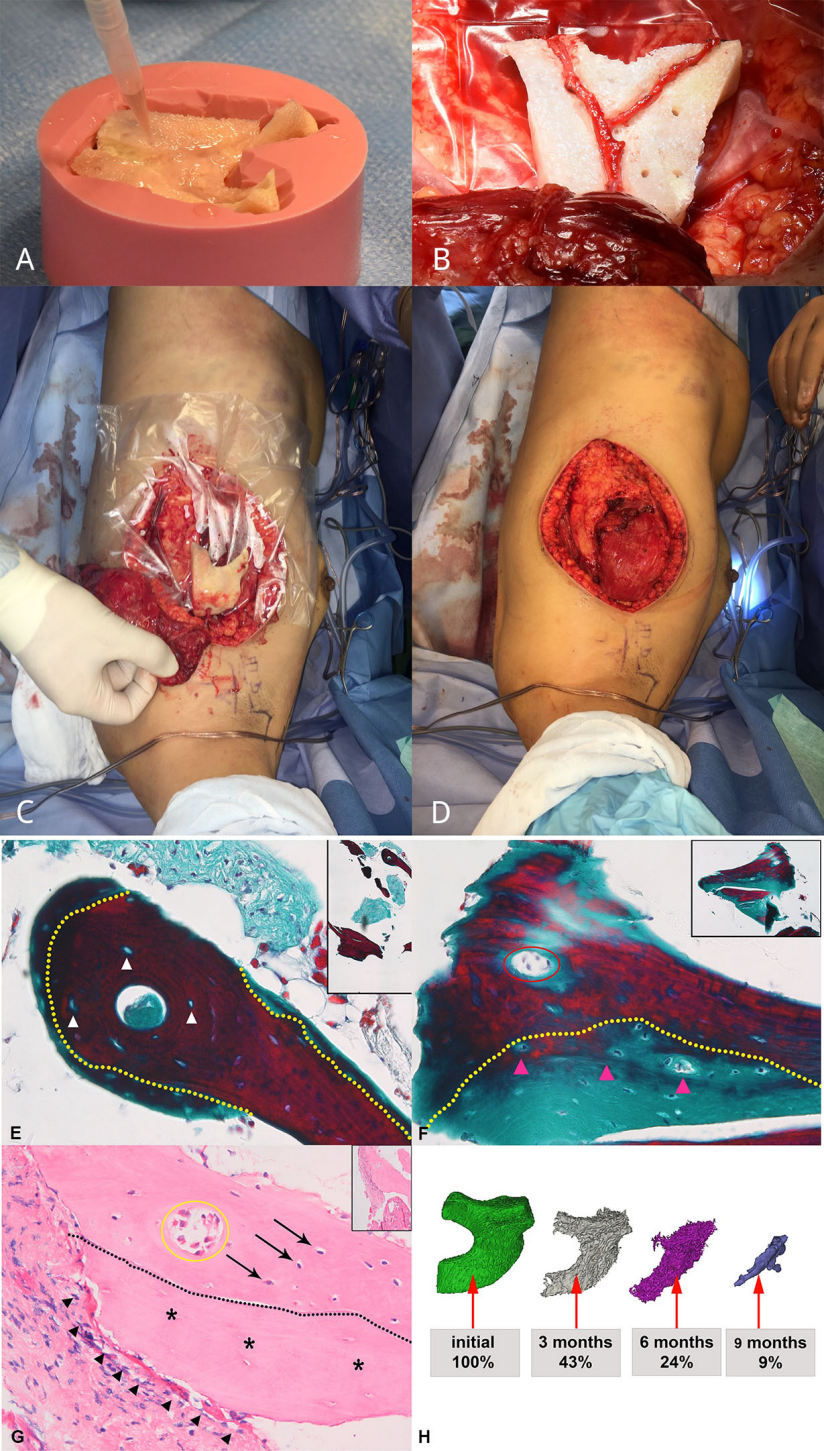
Graft prefabrication and histological analysis (bone biopsy at moment of transfer at week 32) and volume assessment over time. **(A)** 3D scaffold of devitalized bone manufactured to match the patient’s defect size and shape and seeded with SVF cells and BMP-2. **(B)** Ectopic implantation with serratus AV bundle. **(C, D)** Construct with vascular pedicle before and after being wrapped in split latissimus muscle. **(E, F)** High magnification of representative figures of bone biopsy after staining with Masson Trichrome. The Tutoplast^®^ scaffold is characterized by purple staining, representing mature bone and cellular lacunae (white arrowheads), showing devitalized bone tissue. Newly formed bone tissue, represented by light green color, is deposited on the Tutoplast^®^ scaffold and contains nuclei (pink arrowheads). The yellow dashed line delineates the original scaffold material and apposition of newly formed bone. A vessel (red circle) demonstrates that the scaffold is vascularized. **(G)** Overview figure shows appositional bone growth on the Tutoplast^®^ scaffold (asterisk). Osteocytes (arrows) are visible in the newly formed bone. The proportion of scaffold vs. new bone formation is close to 50:50. A blood vessel is present within the newly formed bone (yellow circle). Osteoclasts (full arrowheads) fringe the Tutoplast^®^ scaffold (asterisks), which shows clear signs of degradation at site of interaction. There is no major osteoclast infiltration at the level of the newly formed apposed bone and no sign of degradation visible. **(H)** CT-reconstruction and volume calculation show volume decrease over time.

The patient’s treatment consisted of a two-step reconstructive plan, namely, an ectopic implant prefabrication (**Figure 1D**), followed by its orthotopic transfer (**Figure 1E**). A three-dimensional scaffold was manufactured, based on the patient’s computed tomography (CT) data of the defect site and contralateral side by computer-aided design and computer-aided manufacturing (CAD-CAM) techniques. As scaffolding material, allogenic and decellularized cancellous bone (Tutoplast^®^, Tutogen Medical, Neunkirchen, Germany) with a porosity of approximately 60% was used. The scaffold, with a total volume of 27.6 cm^3^, was designed as a set of four pieces, which could be intraoperatively assembled and fixed with preheated absorbable pins, leaving space for a central tunnel to allow for the intraoperative insertion of an AV bundle (**Figure 1E**, **Figure 2B**).

All surgeries were performed under general anesthesia. To collect stromal vascular fraction (SVF) cells, a hand-assisted abdominal liposuction was performed and a final volume of 320 ml of sedimented fat was harvested. SVF cells were intraoperatively isolated with an automated device (Celution 800/CRS System, Cytori Therapeutics Inc., UK) and counted with a NucleoCounter NC-200™ (ChemoMetec, Denmark), leading to a total number of 106 × 10^6^ cells with a viability of 76.8 +/- 3.2%. After assembling the four Tutoplast pieces with pins, the scaffold was cellularized by gentle loading of 105 × 10^6^ SVF cells, resuspended in 8.5 ml of fibrin gel (Tisseel^®^, Baxter, AT) in the presence of 60 µg/ml recombinant human bone morphogenetic protein-2 (BMP-2, Infuse^®^, Medtronic, CH) (**Figure 2A**). Following SVF cell seeding, a distally ligated arteriovenous (AV) bundle (serratus branch of the thoracodorsal vessels) was surgically inserted in the central scaffold tunnel for axial prevascularization (**Figure 2B**). The whole construct was wrapped in a split latissimus dorsi muscle flap and placed under the patient’s left breast (**Figures 2C, D**). Total surgery time was 6 h and 30 min, while flap raise including vessel dissection and ectopic construct preparation (fixation of the scaffold parts, SVF isolation, seeding of the scaffold) was performed simultaneously.

Vascularization of the construct was analyzed by perfusion magnetic resonance imaging (MRI) after 1 and 6 weeks. Bone metabolism inside the construct was assessed with single-photon emission computed tomography (SPECT) after 6 weeks.

After 8 months and proven neovascularization with manifest bone metabolism as seen on the SPECT scan, the composite graft was harvested and positioned into the recipient site, while a punch biopsy (4 × 5 mm) was taken from the central volume for histological analysis. The thoracodorsal vessels were dissected free, cut, and anastomosed in an end-to-end fashion to the left facial artery and vein. Graft vascularization after transfer was intraoperatively assessed by fluorescent indo cyanine green (ICG) visualization (VisionSense, Philadelphia). The donor site was then settled by primary closure. Postoperative monitoring at the intensive care unit (ICU) was uneventful. Total surgery time was 8 h 18 min. The patient was hospitalized for 7 days. Suction drains at the donor site were removed on the third postoperative day. Antibiotic therapy with amoxicillin/clavulanic acid for a total of 5 days was administered perioperatively.

Regular follow-up appointments were scheduled at 2 and 6 weeks as well as 3, 6, and 12 months post-reconstruction. Final imaging by CT scan and logopedic evaluation to assess speech and swallowing function were conducted 24 months after orthotopic transfer.

## Results

### Ectopic Implant Prefabrication

The patient had an uneventful course after ectopic graft placement in December 2016, without intraoperative complications such as major bleeding or pedicle damage. The patient did not report any implant-related discomfort, and neither hematoma nor infection was noted.

**Figure 3:**
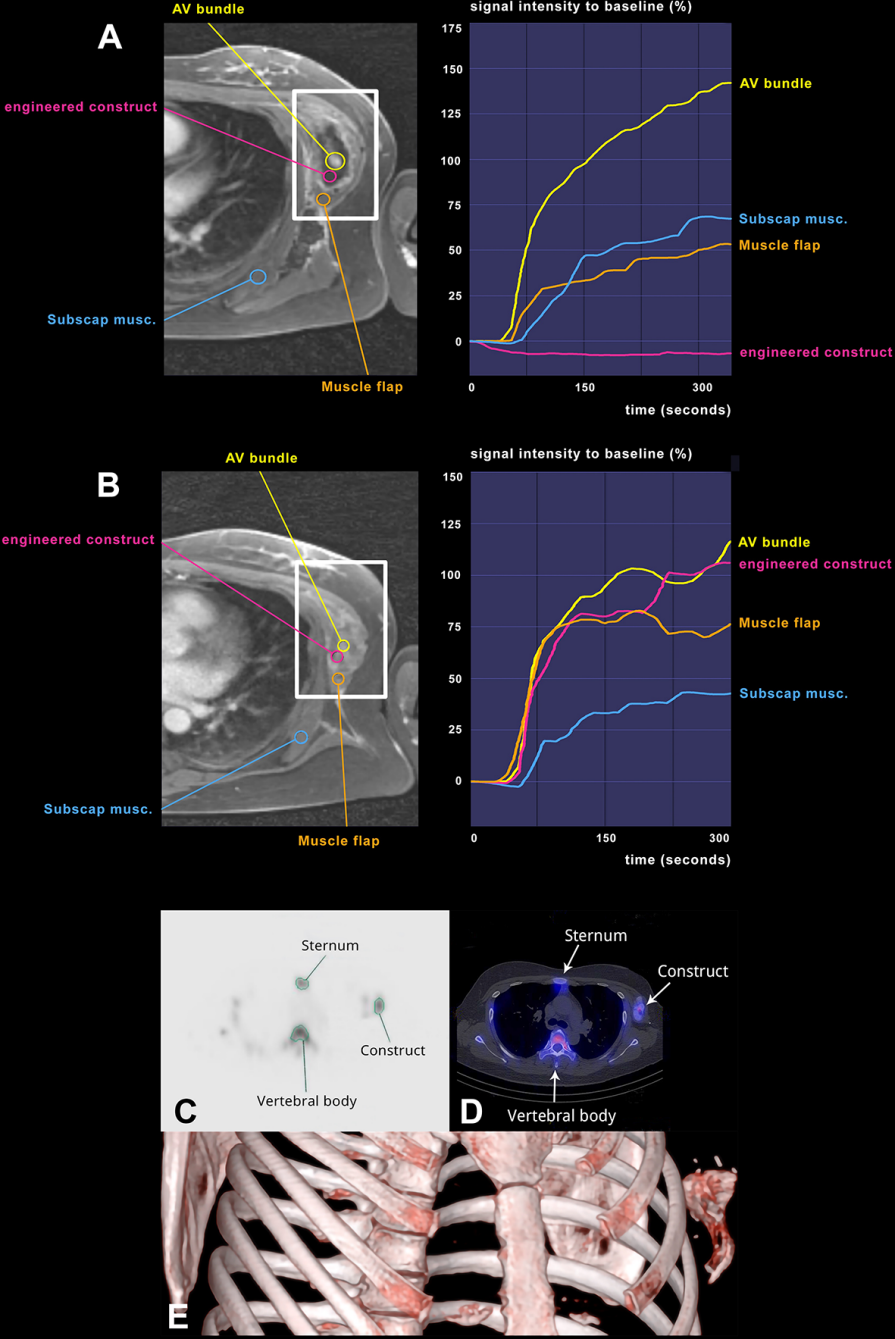
Longitudinal assessment of vascularization and bone formation. Transversal images of a golden-angle radial sparse parallel (GRASP) MRI showing the perfusion of the engineered construct adjacent to the left ribcage over time as well as corresponding signal-time curves at 1 week **(A)** and at 6 weeks **(B)**. The Maxilla construct is highlighted by white rectangles. Signal intensity in relation to t = 0 (injection of contrast agent) on the Y-axis, time in seconds on the X-axis. Violet region of interest (ROI) located in the construct, blue ROI located in the left M. subscapularis *in situ* and yellow ROI encompassing the AV bundle. Orange ROI indicates M. latissimus dorsi flap covering the construct. M. subscapularis was used as a reference for physiological vascularization in this area. Planar images **(C)** and SPECT and SPECT/CT images **(D)** after 6 weeks of the bone scintigraphy, showing strong DPD uptake in the construct, indicating bone turnover and vitality of the construct. In a region-of-interest (ROI) analysis of the maxilla construct at its largest diameter in comparison to the sternum (as a bone with similar structure/size). **(E)** VRT (volume rendering technique) from the SPECT illustrates the location of the prefabricated construct.

Vascularization of the construct was assessed during ectopic graft development after 1 week (**Figure 3A**) and 6 weeks (**Figure 3B**) by dynamic contrast-enhanced MR perfusion imaging. The AV bundle proved to be patent, providing intrinsic perfusion to the construct. The latissimus dorsi muscle wrap was viable, with a perfusion pattern comparable or superior to the subscapularis muscle, chosen as a reference for physiological perfusion in this anatomic region. At both time points, the subscapularis muscle, the latissimus muscle, and the AV bundle showed a normal, steadily increasing perfusion over time. When focusing on the engineered construct, no signal increase after injection of the contrast agent was observed after 1 week, whereas a steep increase in signal intensity was visible after 6 weeks, demonstrating functional internal vascularization.

Bone metabolism of the construct was assessed after 6 weeks by SPECT/CT (**Figures 3C–E**). Active 3,3-diphosphono-1,2-propanodicarboxylic acid (DPD) uptake in the construct revealed traits of viable bone tissue (maximum counts: 112), in the range between the sternal bone (maximum counts: 95) and the highly compact thoracic vertebral body (maximum counts: 154), used as reference structures of similar size and position (**Figures 3C, D**). Bone biopsy at the phase of construct transfer, 32 weeks after ectopic implantation, morphologically confirmed *de novo* bone formation (**Figures 2E–G**). Masson Trichrome staining showed a clear distinction between the Tutoplast^®^ scaffold material, consisting of devitalized bone, and newly deposited bone matrix. The latter was less dense and less mature, as shown by the green color and reduced lamellar structure. The deposited bone contained stained cell nuclei as sign of living tissue, clearly distinguishable from the devitalized bone of the scaffold, where cells were visible as empty lacunae. Vascularization of the construct was histologically verified by piercing vessels in the scaffold material (**Figures 2F, G**). Osteoclasts were found around the Tutoplast^®^ scaffold, which displayed morphological signs of active resorption. Within the newly formed, apposed bone, there was no major osteoclast infiltration and no signs of degradation. Quantitative histomorphometry of the biopsy specimen indicated an average of 30.2% (SD = 10.3) of newly formed bone over the total bone area.

Mineralized tissue volume in the construct was assessed by CT scan at 3, 6, and 9 months postoperatively (**Figure 2H**). Progressive resorption of the mineralized mass was observed, down to 43%, 24%, and 9% of the initial scaffold volume.

### Orthotopic Transplantation

**Figure 4:**
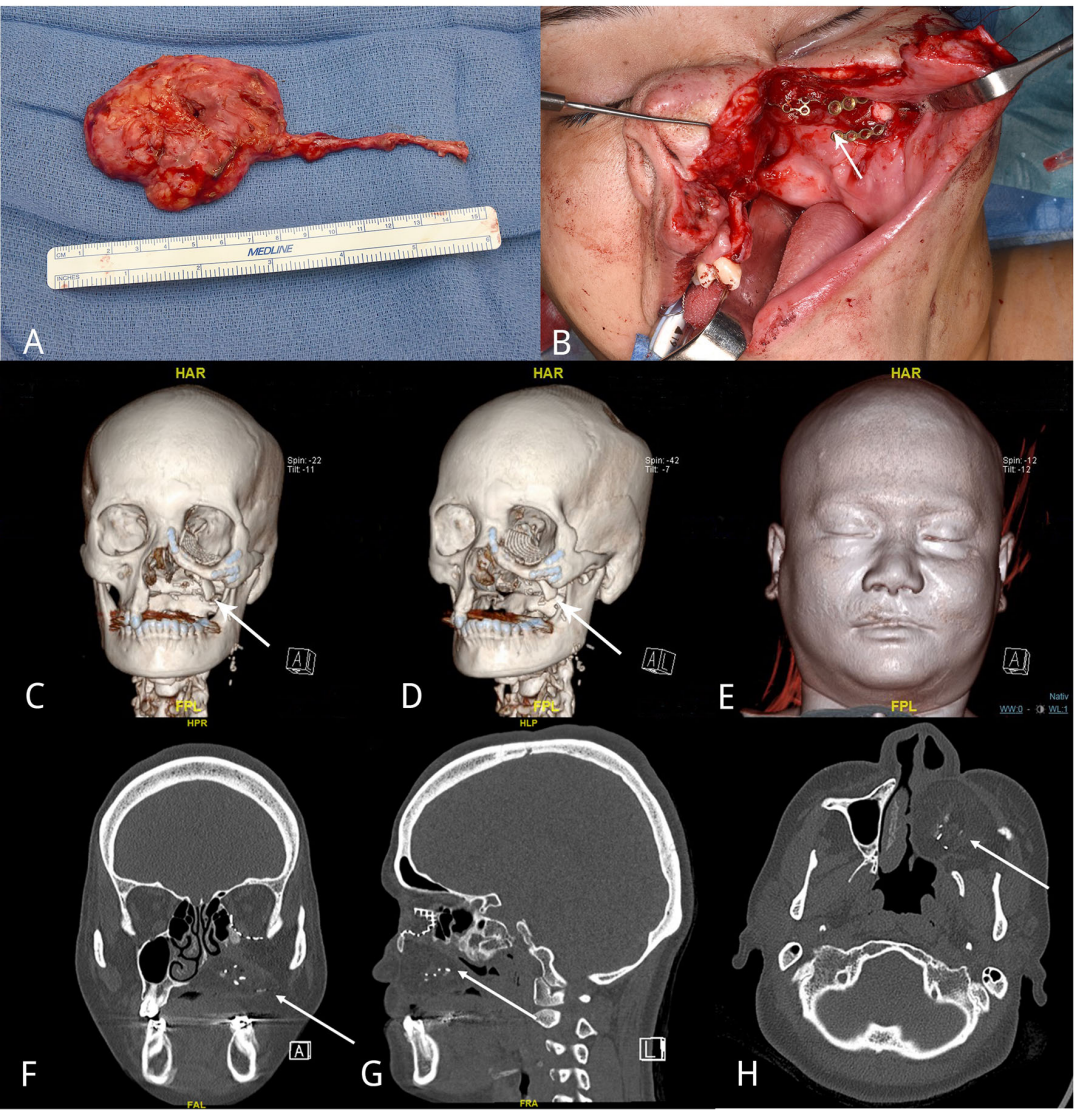
Orthotopic graft transfer and follow-up imaging. Free tissue transfer of the engineered bone-soft tissue composite was performed 9 months after the first step of prefabrication. **(A)** Harvesting of the composite graft, consisting of wrapped latissimus dorsi muscle and the engineered vital bone germ and the AV-bundle. **(B)** Transfacial incision. Exposure of the defect after removal of PEEK implant. **(C, D)** Tabula externa (white arrows) struts served as a substitute for missing bone parts in order to reconstruct the infraorbital rim. **(E)** 3D rendering with symmetric soft tissue coverage. Follow-up CT-imaging 24 months after the orthotopic transfer of the graft from **(F)** coronary, **(G)** sagittal, and **(H)** transverse views. The arrows point to the reconstructed bone tissue present after 24 months.

After 32 weeks, despite advanced resorption, the ectopically engineered composite graft was transferred into the maxillary defect. It was anastomosed to the facial artery and external jugular vein as a free tissue transfer (**Figures 4A, B**). The graft was augmented with calvarial bone struts from the patient’s left parietal region in order to compensate for the loss of bone volume from the initial design. The postoperative course was uneventful. After one night in the ICU, the patient was transferred to the general ward and dismissed after 7 days. Wound healing and flap integration were satisfactory.

After additional 24 months, oral rehabilitation was evaluated with a clinical assessment of the swallowing and speech function as well as a palatogram and myometric measurement of the lips, tongue, masseter muscle, and mentalis muscle by a speech therapist. The patient yielded a near-to-normal oropharyngeal function with normal speech. She was able to eat an unrestricted diet, presented no ectropion, enophthalmos, or diplopia corresponding to normal globe position and function. No microstomy was observed, and oral competence was restored including normal tongue movement, mouth opening, and oral and lip sensation (**Table 1**). Although the patient reported increased trapezius and suboccipital muscle tonicity and slight drooling on the left side, facial symmetry was achieved with aesthetic satisfaction, based on self- and clinical assessment. The CT scan 24 months after orthotropic transfer showed a resorption of the construct with a remaining bone core (**Figures 4C–H**).

**Table 1:** Comparison of the functional outcome between standard of care with autologous tissue transfer and experimental procedure.

Outcome measures	Experimental procedure (described case)	Standard procedure (%) [Cordeiro PG (16)]	Standard procedure (%) [Moreno MA (17)]	Standard procedure (%) [Sweeny AR (18)]
Speech				
Normal	✓	50	47.5	
Nearly normal	x	34.1	40	
Intelligible	x	13.6	7.5	
Unintelligible	x	2.3	5	
Diet				
Unrestricted	✓	52	55	
Soft	x	42	35	
Liquids	x	6	5	
Feeding tube	x	2	5	
Globe Position and function				
Normal	✓	23.8		
Dystopia	x	4.8		
Diplopia	x	19		8
Enophthalmos	x	4.8		
Ectropion	x	47.6		50
Epiphora	x			29
Exposure keratopathy	x			25
Lagopthalmos	x			16
Fistula	x			8
Midface deformity	x			4
Oral competence				
Yes	✓	91.7		
No	x	8.3		
Drooling	✓			
Microstomia				
Yes	x	25		
No	✓	75		
Aesthetic results				
Excellent	✓	58.6		
Good	x	35.7		
Fair	x	5.7		
Poor	x	0		

**Figure 5:**
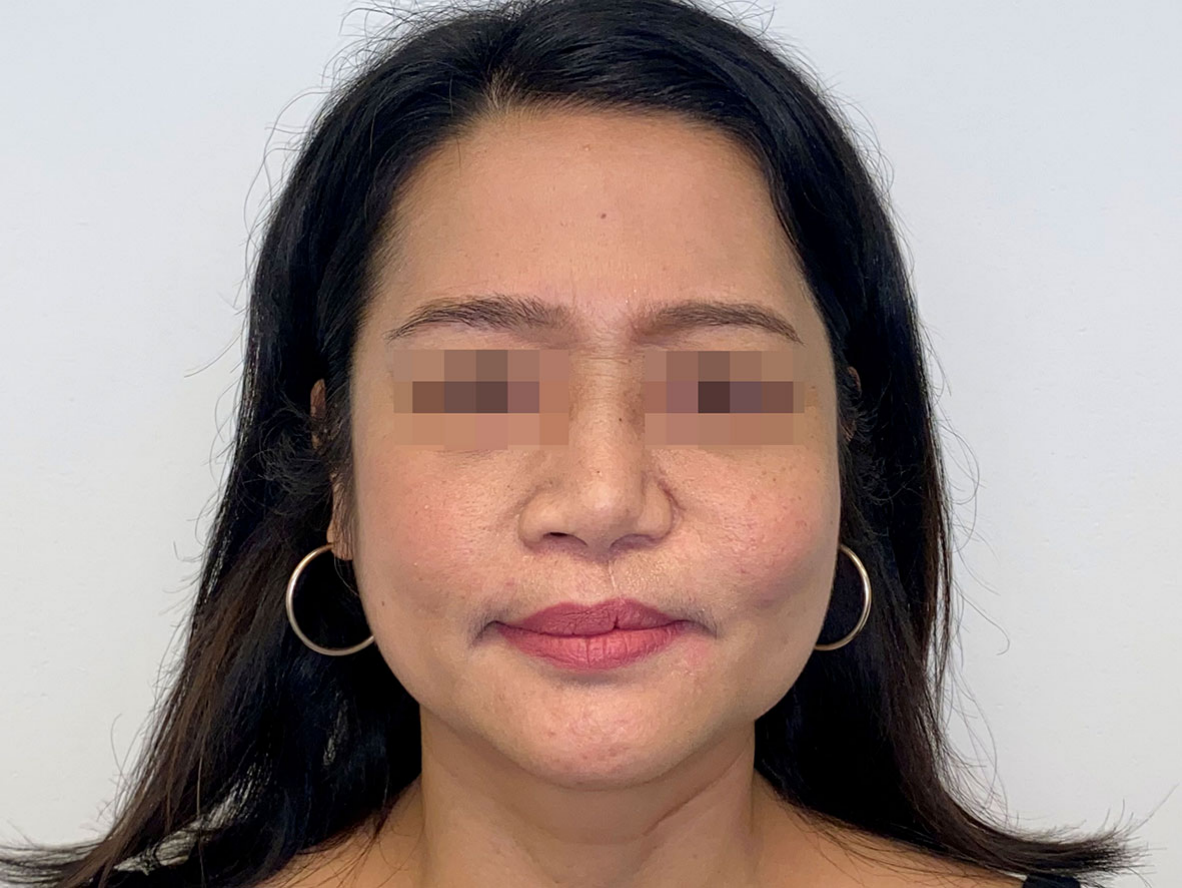
Photographic documentation after reconstruction shows symmetrical and aesthetically pleasing result.

Symmetry and subjective aesthetic outcome were assessed after reconstruction (**Figure 5**).

## Discussion

This case describes the unique combination of bone tissue engineering, ectopic prefabrication, and microvascular free-flap strategies for the reconstruction of a highly complex defect of the left hemimaxilla in a young female patient. With this concept, we were able to create a prefabricated, composite maxilla substitute providing both bone and a soft tissue interface, thus avoiding the use of a non-autologous structural support material, such as a titanium cage, and the morbidity associated with autologous vascularized bone tissue transfer.

It is known that midface defects, especially of the maxilla, can have a substantial functional impact. Conventional reconstructive options, including freely transferred or local tissue flaps, typically fail to provide a sufficient anatomical and functional coverage for defects involving the hard and soft palate, as well as the orbital floor (19, 20). This leads to inadequate mastication, food processing, and lack of separation between the oral and nasal cavities. Traditionally, combinations of non-vascularized bone grafts with myocutaneous free flaps as well as free osteomyocutaneous flaps have been used for the coverage of these defects, all of them with specific advantages and disadvantages. Several chimeric free flaps have been described from the fibula or scapula with skin island. Extensive defects involving the palate and orbital floor, as in the patient described, have a better functional outcome and quality of life if reconstructed with free flaps, compared to prostheses. Still, this is a valuable option in mild to moderate-sized palatal defects or in elderly, multimorbid patients (21, 22). Free flaps in general inherently cause a donor site defect and associated complications. In free fibula bone flaps, which are often used in maxillofacial reconstruction, perioperative donor site complications occur in about one-third of cases and long-term morbidities of 17% have been described, including leg weakness, ankle instability, hallux contracture, and decreased ankle mobility (23). Other osteocutaneous flaps, such as the iliac crest flap, can lead to a sensory deficit (up to 27%), chronic pain (8%-26%) (24, 25), or impaired gait and reduced range of hip motion (25%) (26). For the radial forearm osteocutaneous flap, wound breakdown with tendon exposure is known to occur in 5%-46% (27, 28), whereas fracture of the residual radius occurs in 0%-18% (28–30) and chronic pain in 16.7% (31).

To avoid harvesting large amounts of autologous bone tissue, we decided to combine strategies of tissue engineering with approaches of plastic and reconstructive surgery, implementing a “regenerative surgery” paradigm (32). The bone-forming capacity of the engineered graft was based on three traditional osteogenic principles (33), namely, (i) osteoconduction, provided by the allogenic, devitalized scaffolding material, (ii) osteogenesis, through the patient’s own osteoprogenitor cells derived from the intraoperatively gained adipose stromal vascular fraction (SVF), and (iii) osteoinduction, through the delivery of BMP-2. The intraoperative tissue engineering approach was substantiated by previous preclinical and clinical studies (4, 5) and bypassed complex, time-consuming and costly *in vitro* cell culture. The engineered graft, ectopically prefabricated with an associated AV bundle, achieved efficient vascularization and bone formation within a confined space. Moreover, its composite nature, including an interface with soft tissues, enabled to restore the patient’s anatomical and functional deficit, to provide support to the eye globe, to obliterate the communication between the orbit and the nasopharynx, and to reconstruct the palatal surface.

**Table 1** shows in detail the clinical advantages of the developed technique as compared to the expected outcome following the standard of care described by Moreno (17), Cordeiro (16), or Sweeney (18). Moreover, in contrast to previously described cases of tissue engineering-based mandibular defect reconstructions, we were able to avoid foreign material (titanium cage), as used by Warnke et al. and Wiltfang et al. (34, 35). By doing so, we avoided not only the postoperative risk of developing an implant-associated infection which, for titanium implants, is reported to be around 7% but also the life-long risk of later hematogenous infection of the implant (36) and the so-called foreign body reactions, where the implant is encapsulated and cannot be integrated with the surrounding tissue (37).

In contrast to previous studies, we used SVF cells from adipose tissue instead of bone-marrow mesenchymal stromal cells (BM-MSC). Adipose stromal cells have recently emerged as a viable source for clinical applications, because of their abundance and easy access. When compared to BM-MSC, SVF cells do require an osteogenic priming, here offered by the BMP2 delivery, but display higher resistance to hypoxia-induced apoptosis and oxidative stress-induced senescence and have more potent proangiogenic activity (38–41).

The main limitation of the developed procedure was a significant resorption of the original scaffold material during the construct prefabrication, so that a sufficient bone stock could not be maintained for dental implants, representing the ultimate solution for mastication and phonation. One possible explanation for the observed resorption might be due to an effect of the supplied BMP-2. Besides their osteoinductive properties, BMPs are known to invoke a seemingly dose-dependent allograft resorption mediated by osteoclasts. Pradhan et al. reported that BMP-2 treatment of a bone graft might cause a higher non-union rate compared with non-treatment, which was attributed to an aggressive bone-resorptive phase prior to osteoinduction (42). Similarly, Vaidya et al. showed that BMP-2-treated bone grafts for spinal fusion lost their original height and structure, likely due to activated bone resorption (43). In addition, Seeherman et al. recently reported that treatment with BMP-2 in a primate bone defect model increased the size of the defect and the number of osteoclasts, so that the expected bone formation was preceded by bone resorption (44). These clinical studies are consistent with experimental evidences that BMP-2 and BMP-7 may reduce bone size by directly or indirectly activating osteoclasts (45). Other possible explanations for the bone resorption observed in the present case could be related to the missing mechanical load during the ectopic prefabrication phase (46) or to the biological influence of the muscle tissue in direct contact with the construct. In fact, it was reported that in calcium sulfate/apatite bone substitutes with direct contact to muscle, the calcium sulfate phase was resorbed after 6 weeks and the hydroxyapatite content decreased significantly over time (47).

Further studies are therefore required to identify the underlying cause of bone resorption and to possibly counteract it. For example, combined anti-resorptive therapies, such as bisphosphonates, have been preclinically validated in a variety of bone repair models, especially to reduce BMP catabolic effects (48–51), and may be considered in future developments. More bone mass would allow the patient to receive tooth implants after the primary procedure and without need of additional bone transfer to the defect site.

Despite this limitation, the engineered graft and its surgical implementation achieved a separation between the oral and nasal cavities in the setting of a critical maxilla defect and provided structural support to the orbital floor. This was of substantial benefit to the patient, without drawbacks of free vascularized autologous bone grafting procedures or foreign body implantation. The clinical case thus represents a proof of principle for a “regenerative surgery”-based prefabrication concept and warrants consideration for reconstruction of complex composite defects in functionally and aesthetically highly demanding areas.

## Data Availability Statement

The original contributions presented in the study are included in the article/supplementary material. Further inquiries can be directed to the corresponding author.

## Ethics Statement

The studies involving human participants were reviewed and approved by Swissmedic, under exceptional permission (SBH 16-0172) of the ethical committee (EKNZ). The patients/participants provided their written informed consent to participate in this study.

## Author Contributions

TI and AH were responsible for the conception and design, acquisition of data, analysis and interpretation of data, and writing and revision of the manuscript. TI, AH, DS, AL, AK, FT, CK, and CJ performed the surgery. AL, RO, AK, FT, and FS contributed to the analysis and interpretation of data and revision of the manuscript. JB conceived and designed the analysis, collected the data, and contributed data or analysis tools. TW and FK contributed to the conception and design, acquisition of data, and analysis and interpretation of data. AS, DS, and IM provided senior advice, counseling, writing support, and revision of the manuscript. AW and SM provided conception and design and regulatory aspects. All authors contributed to the article and approved the submitted version.

## Funding

This work was supported by the Propatient Forschungsstiftung, University Hospital Basel.

## Conflict of Interest

The authors declare that the research was conducted in the absence of any commercial or financial relationships that could be construed as a potential conflict of interest.

## Publisher’s Note

All claims expressed in this article are solely those of the authors and do not necessarily represent those of their affiliated organizations, or those of the publisher, the editors and the reviewers. Any product that may be evaluated in this article, or claim that may be made by its manufacturer, is not guaranteed or endorsed by the publisher.
